# Fetal Midgut Volvulus with Meconium Peritonitis Detected on Prenatal Ultrasound

**DOI:** 10.1155/2018/5312179

**Published:** 2018-05-03

**Authors:** Emanuelle J. Best, Cecelia M. O'Brien, Wendy Carseldine, Aniruddh Deshpande, Rebecca Glover, Felicity Park

**Affiliations:** ^1^Maternity and Gynaecology, John Hunter Hospital, New Lambton Heights, NSW, Australia; ^2^Maternal Fetal Medicine Unit, John Hunter Hospital, New Lambton Heights, NSW, Australia; ^3^Department of Paediatric Surgery, John Hunter Children's Hospital, New Lambton Heights, NSW, Australia; ^4^Neonatal Intensive Care Unit, John Hunter Children's Hospital, New Lambton Heights, NSW, Australia

## Abstract

**Background:**

Fetal volvulus is a rare, yet life-threatening condition that requires skilful diagnosis and management. Volvulus occurs when bowel loops become twisted and the twisting of the mesenteric artery leads to congestion, impaired venous return, and bowel necrosis.

**Case Description:**

We present a case of fetal ileal volvulus suspected on third trimester ultrasound, complicated by premature labour, small bowel necrosis, and meconium peritonitis. Progressive dilatation and decreased peristalsis of echogenic bowel were noted in the early part of the third trimester. Daily surveillance ultrasound was performed and spontaneous labour occurred at 32 weeks' gestation. A proactive postnatal approach guided by prenatal sonographic findings allowed prompt treatment and an urgent laparotomy was performed for an ileal volvulus with necrosis and meconium peritonitis. A segment of small bowel volvulus was resected and an end-to-end anastomosis was performed with uneventful recovery.

**Discussion:**

Clinically signs of fetal midgut volvulus are not pathognomonic, such as intestinal dilatation, abdominal mass, ascites, peritoneal calcifications, or polyhydramnios; thus, the diagnosis is often challenging. Complications reported in the literature include perforation and haemorrhagic ascites, which may lead to anaemia, hypovolemia, heart failure, and fetal demise.

**Conclusion:**

This case highlights the importance of assessing the fetal bowel as a part of routine third trimester ultrasound. The case describes the complexity of diagnosis in the fetus, important considerations along with multidisciplinary team approach to management.

## 1. Introduction

Fetal volvulus is a rare, yet life-threatening condition that requires skilful diagnosis and management. Volvulus occurs when bowel loops become twisted around the mesenteric artery or its branches [1]. The twisting of the mesenteric artery leads to intestinal and vascular congestion, leading to impaired venous return and bowel necrosis [2].

The diagnosis of this condition can be challenging, as the antenatal clinical presentation is often nonspecific. From the literature, clinical symptoms include decreased fetal movements, increased fundal height due to polyhydramnios, or a nonreassuring cardiotocography (CTG) [1]. Ultrasound findings that may increase suspicion of fetal midgut volvulus may include dilated bowel loops, intraabdominal calcifications, abdominal mass, polyhydramnios, gastric dilatation, and ascites. Doppler studies can demonstrate an elevated peak systolic velocity in the middle cerebral artery due to severe fetal anaemia, secondary to haemorrhagic ascites. Complications of fetal volvulus that are reported in the literature include bowel perforation, hypovolemia, heart failure, pleural and pericardial effusions, and fetal demise [2–5].

This report details the management of fetal segmental ileal volvulus diagnosed on ultrasonography in the 3rd trimester.

## 2. Case Presentation

**Figure 1:**
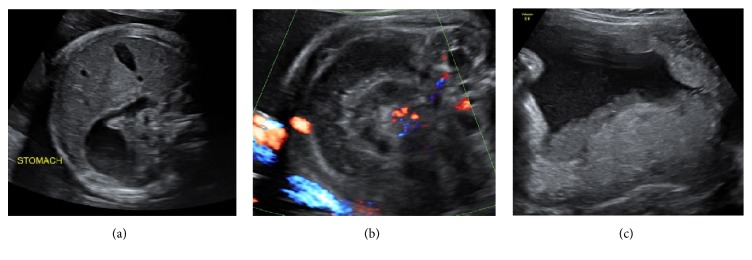
Ultrasound images of the key antenatal findings: (a) dilated appearing stomach with relatively normal duodenal diameter, not consistent with duodenal atresia; (b) concentric small bowel visible around the twisted mesenteric pedicle (whirlpool sign) and the superior mesenteric vein malpositioned on the left of the artery; and (c) dense sediment noted in the amniotic fluid, which was noted to be bile at the time of delivery.

A 22-year-old woman, gravida 3, para 2, was referred to our tertiary centre at 31 weeks and 5 days' gestation due to a nonreassuring antenatal CTG with mild fetal tachycardia and decreased fetal movements. Initial ultrasound examination showed a moderately dilated fetal stomach, distended bowel loops, and aperistalsis (Figure 1). Doppler studies were normal, there was no evidence of fetal anaemia, and the amniotic fluid index was within normal range.

Further investigations included an amniocentesis, which demonstrated a normal fluorescence in situ hybridization (FISH) for chromosomes 21, 18, and 13 in a female fetus. A single nucleotide polymorphism (SNP) microarray showed a 0.56 Mb deletion at 16q23.3 loci of uncertain clinical significance, which was not parentally inherited. Maternal viral serology, Kleihauer, and cystic fibrosis testing were negative. Fetal echocardiogram confirmed a small pericardial effusion in an otherwise normal study.

**Figure 2:**
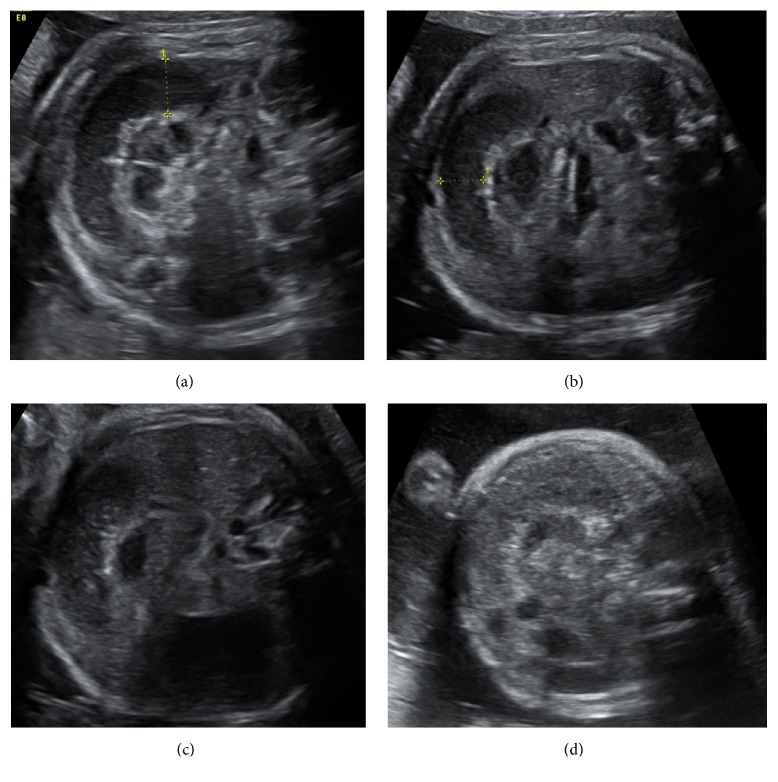
Ultrasound images showing progressive changes within the fetal bowel over time. (a) At 31^+6^ weeks, echolucent bowel contents with a diameter of 14 mm. (b) At 31^+6^ weeks, a loss of clear bowel wall border. (c) At 32 weeks, increasing echogenic particles within the bowel lumen. (d) At 32^+1^ weeks, bowel contents appear echogenic.

Daily ultrasound surveillance showed persisting bowel dilatation up to 18 mm in diameter, with increasing echogenicity noted over time, with minimal peristalsis and loss of definition in the bowel wall, indicating oedema as shown in Figure 2. The coffee-bean and whirlpool signs were both evident; the latter is shown in Figures 1 and 2. At 32 weeks' gestation, corticosteroids were administered for fetal lung maturity in light of the progressive bowel dilatation and further discussions were being undertaken with continued multidisciplinary team input.

At 32 weeks and 2 days' gestation, labour began spontaneously. A live female infant was delivered vaginally with a birthweight of 2450 g (greater than 97th percentile for gestation). Apgar scores were 9^1^ and 9^5^ without significant requirement for resuscitation. The arterial cord gas showed a pH of 7.28 and lactate of 5.4 mmol/L, and the venous cord gas gave a pH of 7.44 and lactate of 3.8 mmol/L. The infant was admitted to the Neonatal Intensive Care Unit (NICU) at 15 minutes of age for respiratory support with continuous positive airway pressure (CPAP).

**Figure 3:**
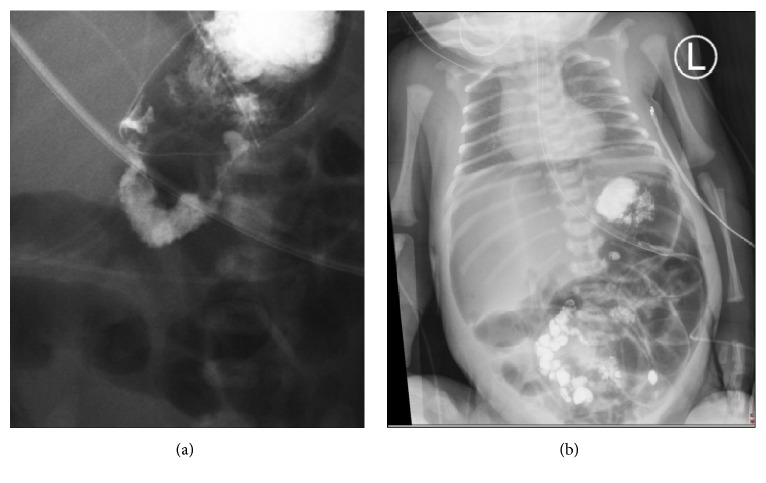
Radiographic images from day 1 and day 2 postnatally. (a) Contrast study performed on day 1 of life excluding malrotation and (b) large pneumoperitoneum evident on abdominal X-ray on day 2 of life.

**Figure 4:**
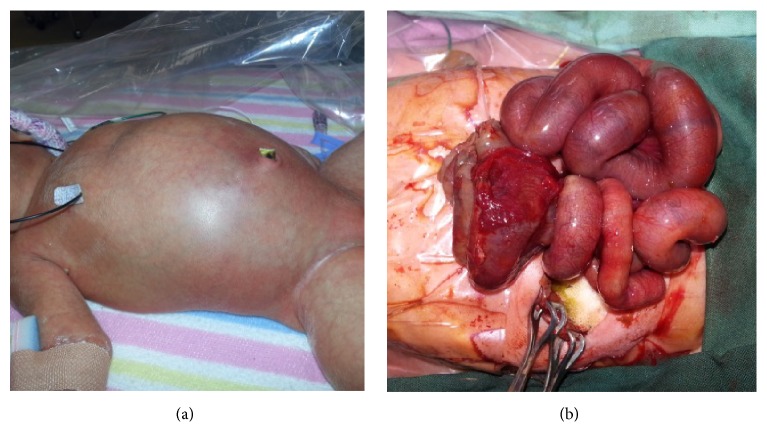
Clinical photos: (a) clinical appearance of infant on day 2 of life with erythematous, distended abdomen, and (b) in operating theatres ileal volvulus visible with a necrotic segment on the left side.

On day 1 of life, the baby remained well with no abdominal signs and a contrast swallow was performed, which did not show obvious malrotation (Figure 3(a)). The baby was kept fasted and remained under close surveillance for clinical signs or symptoms of distress. An abdominal X-ray on day 2 showed a pneumoperitoneum (Figure 3(b)). An urgent laparotomy was then performed, with findings of an ileal volvulus with meconium peritonitis (Figure 4). This required resection of the necrotic small bowel volvulus segment with a successful end-to-end anastomosis achieved.

On day 2, the infant's postoperative haemoglobin was 101 g/L and 1 unit of packed red blood cells was transfused which increased the level to 129 g/L. The postoperative course was otherwise uncomplicated with discharge home on day 7. Surgical follow-up occurred at thirteen weeks chronological age (five weeks corrected age), where the infant was found to have good scar healing and a soft nondistended abdomen. Feeding and weight gain were appropriate along with a reported normal bowel function.

On histopathological examination, the resected small intestine showed established necrosis with organisation including fibrous replacement of necrotic muscularis propria and secondary adhesions. Fibroblastic proliferation was also seen within the mesentery, which showed focal necrosis and recanalisation of mesenteric vessels. These findings are consistent with a clinical diagnosis of volvulus. The necrosis and organisation were well established at the time of resection on postnatal day 2, which is consistent with antenatal occurrence. There was no focal lesion found to explain the cause of the volvulus with no evidence of Meckel's diverticulum, tumour, or malignancy.

## 3. Discussion

Our case highlights the clinical benefit of prenatal sonographic findings in prompt postnatal care of fetal volvulus.

The incidence of fetal volvulus is not described in the literature; however, symptomatic neonatal intestinal rotation has been estimated at 1 in 6000 [3]. Small bowel related complications such as atresia, obstruction, and volvulus have a reported incidence of 1 in 1500 and 1 in 3000 [4, 5]. There are three types of volvulus presenting in the prenatal or newborn period, namely, classic type, segmental type, and volvulus without malposition. Firstly, the classic type is defined as malposition of bowel due to clockwise rotation of the small bowel and ascending colon, around the superior mesenteric artery without evidence of any anomaly predisposing to the rotation [6]. Segmental volvulus has been described as twisting of bowel loops due to an anomaly such as meconium ileus (related to cystic fibrosis), atresia, mesenteric defects, duplication or mesenteric cysts, congenital diaphragmatic hernia or abdominal wall defects, or alternatively can be classified as idiopathic [7]. Lastly, volvulus without malposition is a diagnosis of exclusion and is more common in extremely premature or low birthweight infants [6]. It is thought to be due to the insufficient fixation of the intestines due to prematurity. The latter is rare and difficult to diagnose due to the lack of consistent clinical signs, unlike the classic type.

Symptoms and signs of fetal volvulus may include decreased fetal movements, polyhydramnios, nonreassuring CTG, dilated bowel loops, gastric dilatation and ascites on diagnostic ultrasound, or ultrasound evidence of fetal anaemia (middle cerebral artery peak systolic velocity greater than or equal to 1.5 MoM) secondary to haemorrhagic ascites [1]. Complications of fetal volvulus may include bowel perforation, hypovolemia, heart failure, pleural and pericardial effusions, or fetal demise [2, 8–10].

Intestinal obstruction and haemorrhagic ascites can lead to the complication of fetal anaemia, which occurred in 4 (36%) of the 11 case reports in the literature. Three studies from Noreldeen et al., Tan et al., and Kornacki et al. all reported cases of fetal volvulus where ascites was identified on prenatal ultrasound and an elevated MCA PSV was detected, suggestive of fetal anaemia, with the diagnosis of volvulus confirmed on postnatal laparotomy [1, 10, 11]. However, in the case reported by Noreldeen et al., after an emergency caesarean was performed at 31 weeks, the fetus was found to be severely anaemic, requiring resuscitation with endotracheal intubation and a blood transfusion as well as an emergency laparotomy [1]. Steffensen et al. reported a fetal demise occurring in utero at 38 weeks of gestation due to cardiovascular failure from midgut volvulus and severe fetal anaemia, after ultrasound findings of ascites and dilated bowel loops were present from 15 weeks' gestation. The infant was later found to have intestinal atresia and arthrogryposis [2].

The most extensive retrospective case series by Sciarrone et al. was reported recently in 2016 [8]. This included eight cases of fetal volvulus and description of the ultrasound findings that led to the diagnosis [8]. In all eight cases, prominent bowel loops were visualised on prenatal ultrasound. Polyhydramnios was a notable finding in three of the eight cases and ascites was present in two cases. Chao et al. had found that the whirlpool sign, which is a spiral shaped mass made up of dilated bowel loops seen on ultrasound, is a useful indicator of fetal volvulus [12]. This prenatal ultrasound sign was present in eight of the nine cases in their 3-year prospective study and thus reported to have a sensitivity of 89% and specificity of 92%. Conversely, in the case series by Sciarrone et al. the whirlpool sign was visualised in only one of the eight cases [8]. The coffee-bean sign refers to the dilatation of the small bowel with a thin outer layer composed of a single bowel wall layer and a thick inner wall due to double wall thickness of opposed bowel loop. The coffee-bean sign is regarded as a specific sign of volvulus in both radiographic and ultrasonic imaging, and 4 cases have reported this finding in the fetus [8, 9].

Interestingly, three of the eight cases reported in this case series tested positive for cystic fibrosis [8]. The association between cystic fibrosis and fetal volvulus has been described by Durand et al. [7], whereby the malfunction of the transmembrane conductance regulator leads to viscous mucous secretions, which then obstructs the bowel and predisposes to volvulus [7].

The recommended management includes referral to a tertiary centre with neonatal surgical services, multidisciplinary team planning for antenatal, intrapartum, and neonatal care. Continued close surveillance of fetal wellbeing is warranted if delivery is not imminent and prompt surgical review and management in the neonatal period [9–13]. The indications for delivery that have been documented in the literature include persistent significant ascites causing cardiac failure, pulmonary hypertension, pleural or pericardial effusions, or hypovolemic shock [13].

Neonatal management includes close observation and supportive therapy [14]. Serial clinical examination, surveillance for sepsis including inflammatory markers, and coagulopathy and assessment for clinical deterioration are indicated by metabolic acidosis and increasing ventilation requirements [14].

Despite improvements in prenatal diagnosis, there are no studies comparing the outcomes of volvulus diagnosed prenatally versus postnatally [15]. Basu and Burge compared antenatal to postnatal diagnosis in relation to small bowel atresia. The authors found in the 31% of cases that were detected prenatally that these cases had a higher rate and longer duration of total parental nutrition (83% versus 44%) [16]. The severity of bowel obstruction may have contributed to the antenatal detection, therefore influencing the outcomes reported in this study. The clear advantage to prenatal diagnosis relates to preparation of the parents and in utero transfer of patients to tertiary centres for delivery, rather than emergent transfers in the neonatal period and potential separation of the mother and her baby [15]. Further studies are required to further delineate between the outcomes of newborns diagnosed in the prenatal versus postnatal period.

The indications for surgical intervention include pneumoperitoneum, intestinal obstruction, progressive peritonitis, sepsis, and fixed intestinal loop on plain abdominal film [14]. Surgical management at the time of laparotomy in the neonatal period for the treatment of volvulus is ideally primary anastomosis over stoma formation to maximise small bowel length [17].

This case highlights the importance of careful assessment of the fetal bowel in the third trimester ultrasound, the complexities surrounding the diagnosis of fetal midgut volvulus along with the multidisciplinary team approach required for prompt recognition and management.

## 4. Conclusion

With advances in ultrasound technology and its widespread use in the third trimester, the diagnosis of volvulus prenatally may become more common. Timely referral to a tertiary centre with fetal medicine specialists, availability of neonatal intensive care facilities, and paediatric surgical service are paramount to managing this acute bowel complication.

## Abbreviations

CTG: Cardiotocography
AFI: Amniotic fluid index
FISH: Fluorescence in situ hybridization
SNP: Single nucleotide polymorphism
CPAP: Continuous positive airway pressure.

## References

[1] Noreldeen S. A., Hodgett S. G., Venkat-Raman N. (2008). Midgut volvulus with hemorrhagic ascites: a rare cause of fetal anemia.

[2] Steffensen T. S., Gilbert-Barness E., DeStefano K. A., Kontopoulos E. V. (2008). Midgut volvulus causing fetal demise in utero.

[3] Stockmann P. T. (2005). Malrotation.

[4] Best K. E., Tennant P. W., Addor M. C. (2012). Epidemiology of small intestinal atresia in Europe: A register-based study.

[5] Komuro H., Hori T., Amagai T. (2004). The etiologic role of intrauterine volvulus and intussusception in jejunoileal atresia.

[6] Kargl S., Wagner O., Pumberger W. (2015). Volvulus without malposition—a single-center experience.

[7] Durand M., Coste K., Martin A. (2008). Fetal midgut volvulus as a sign for cystic fibrosis.

[8] Sciarrone A., Teruzzi E., Pertusio A. (2016). Fetal midgut volvulus: Report of eight cases.

[9] Jakhere S. G., Saifi S. A., Ranwaka A. A. (2014). Fetal small bowel volvulus without malrotation: The whirlpool & coffee bean signs.

[10] Tan R. M. R., Lee J., Biswas A., Amutha C. (2014). Ascites, anemia and (intestinal) atresia.

[11] Kornacki J., Czarnecka M., Błaszczyński M. (2010). Congenital midgut volvulus associated with fetal anemia.

[12] Chao H.-C., Kong M.-S., Chen J.-Y., Lin S.-J., Lin J.-N. (2000). Sonographic features related to volvulus in neonatal intestinal malrotation.

[13] Kitamura R. K., Midulla P., Mirensky T. (2016). Meconium peritonitis following intestinal atresia: A case report.

[14] de la Hunt M. N. (2006). The acute abdomen in the newborn.

[15] Keskin U., Karasahin K. E., Ozturk M. (2015). Prenatal diagnosis of the acute meconium peritonitis secondary to ileum volvulus perforation: A case report.

[16] Basu R., Burge D. M. (2004). The effect of antenatal diagnosis on the management of small bowel atresia.

[17] Baldassarre M. E., Laneve A., Rizzo A. (2008). A case of fetal midgut volvulus and jejunal atresia: Nutritional support and maintenance of mucosal function and integrity.

